# Influence of hypothermia and subsequent rewarming upon leukocyte-endothelial interactions and expression of Junctional-Adhesion-Molecules A and B

**DOI:** 10.1038/srep21996

**Published:** 2016-02-25

**Authors:** Nicolai V. Bogert, Isabella Werner, Angela Kornberger, Patrick Meybohm, Anton Moritz, Till Keller, Ulrich A. Stock, Andres Beiras-Fernandez

**Affiliations:** 1 Department of Thoracic and Cardiovascular Surgery, University Hospital Frankfurt, Goethe University, Frankfurt/Main, Germany; 2 Department of Anaesthesiology, University Hospital Frankfurt, Goethe University, Frankfurt/Main, Germany; 3 Department of Cardiology, University Hospital Frankfurt, Goethe University, Frankfurt/Main, Germany

## Abstract

Patients with risks of ischemic injury, e.g. during circulatory arrest in cardiac surgery, or after resuscitation are subjected to therapeutic hypothermia. For aortic surgery, the body is traditionally cooled down to 18 °C and then rewarmed to body temperature. The role of hypothermia and the subsequent rewarming process on leukocyte-endothelial interactions and expression of junctional-adhesion-molecules is not clarified yet. Thus, we investigated in an *in-vitro* model the influence of temperature modulation during activation and transendothelial migration of leukocytes through human endothelial cells. Additionally, we investigated the expression of JAMs in the rewarming phase. Exposure to low temperatures alone during transmigration scarcely affects leukocyte extravasation, whereas hypothermia during treatment and transendothelial migration improves leukocyte-endothelial interactions. Rewarming causes a significant up-regulation of transmigration with falling temperatures. JAM-A is significantly modulated during rewarming. Our data suggest that transendothelial migration of leukocytes is not only modulated by cell-activation itself. Activation temperatures and the rewarming process are essential. Continued hypothermia significantly inhibits transendothelial migration, whereas the rewarming process enhances transmigration strongly. The expression of JAMs, especially JAM-A, is strongly modulated during the rewarming process. Endothelial protection prior to warm reperfusion and mild hypothermic conditions reducing the difference between hypothermia and rewarming temperatures should be considered.

Therapeutic perioperative hypothermia during cardiopulmonary bypass (CPB) is standard of care in cardiac- and aortic-surgery since the first practical use in 1958^1^. Reduction of temperature lowers endogenous metabolic rates and tissue oxygen consumption, which enables prolonged operations accompanied with a high risk of ischemia. However, cardiac surgical procedures supported by CPB generate a systemic inflammatory response syndrome (SIRS), which is associated with acute complications and relevant organ dysfunction and even organ failure^2^. The activation of immune response during CPB is a complex process with many triggers. The exposure of circulating blood cells to the surface of an extracorporeal circuit with activation of the complement system^3^ leads to increased cytokine production^4^, such as interleukins (ILs), necrosis factors and bradykinin. Patients undergoing CPB may also show an endotoxemia^5^ with a strongly correlated risk of post-operative infections^6^. Different strategies have been tested to reduce this systemic inflammation, e.g. leukocyte filtration during CPB^7^ or prophylactic application of corticosteroids^8^ with limited success. With the application of therapeutic hypothermia during CPB, patients are not only exposed to the extracorporeal circuit, with the previous mentioned inflammatory response, but also to different temperature expositions during surgery. These temperature conditions in cardiac- and aortic surgery range from mild and moderate hypothermia (28–34 °C), to deep hypothermia conditions (16–18 °C). This range of temperatures should protect not only for ischemic events, but also decrease the systemic inflammatory response. Experimental studies investigated the influence of hypothermia on the production of chemokines and observed a decreasing production of IL-8^9^,^10^ and MCP-1^9^. Looking on the synthesis of pro-inflammatory cytokines, many studies observed a reduction of pro-inflammatory cytokines, e.g. TNF-α, IL-1β^11^, IL-6^12^ and IL-2^13^, whereas anti-inflammatory cytokines like IL-10^11^,^12^,^13^ increase under hypothermic conditions. The production of adhesion molecules is modified during hypothermia and results in a decreased expression of ICAM-1^14^, E-Selectin^15^, VCAM and ELAM^16^. Further clinical and experimental observations have shown that hypothermia is able to modulate the immune response towards an immunosuppressive effect. In contrast, a clinical analysis showed that patients undergoing post-hypothermic rewarming suffer from a higher risk of fever^17^, which could be associated with an over-activation of immune response. Experimental studies have demonstrated that post-hypothermic rewarming increases the production of pro-inflammatory cytokines like IL-6^18^ or IL-8^19^ and leads to an up-regulation of adhesion molecules, e.g. ICAM-1, E-Selectin and VCAM-1^20^.

Therefore, a paradox effect upon inflammatory conditions may be considered. While hypothermia has a protective effect upon inflammation and endothelial modulation, post-hypothermic rewarming could move the immune response towards an activating effect. This pro-inflammatory effect is clinically relevant, as it triggers endothelial leakage and post-ischemic tissue infiltration through transendothelial migration (TEM) of leukocytes, which may result in disadvantages for patients subject to deep hypothermia and posterior rewarming. Endothelial leakage is mediated, among others, by Junctional-adhesion-molecules (JAM). These molecules are mainly located at endothelial or epithelial intercellular junctions and are associated with tight-junctions^21^, being partly responsible for modulation of TEM^21^,^22^.

With an *in-vitro* model we simulated the previous mentioned acute systemic inflammatory response syndrome during CPB and investigated how temperature changes during cell activation and transendothelial migration affect leukocyte recruitment through endothelial cells. Temperature modulation was performed during cell activation, transmigration or during both periods. Furthermore we investigated the expression of JAM-A and JAM-B on endothelial cells after post-hypothermic rewarming to clarify their role in this process.

## Results

### Transmigration assay

#### Hypothermic transmigration reduces transendothelial migration only partially

**Figure 1:**
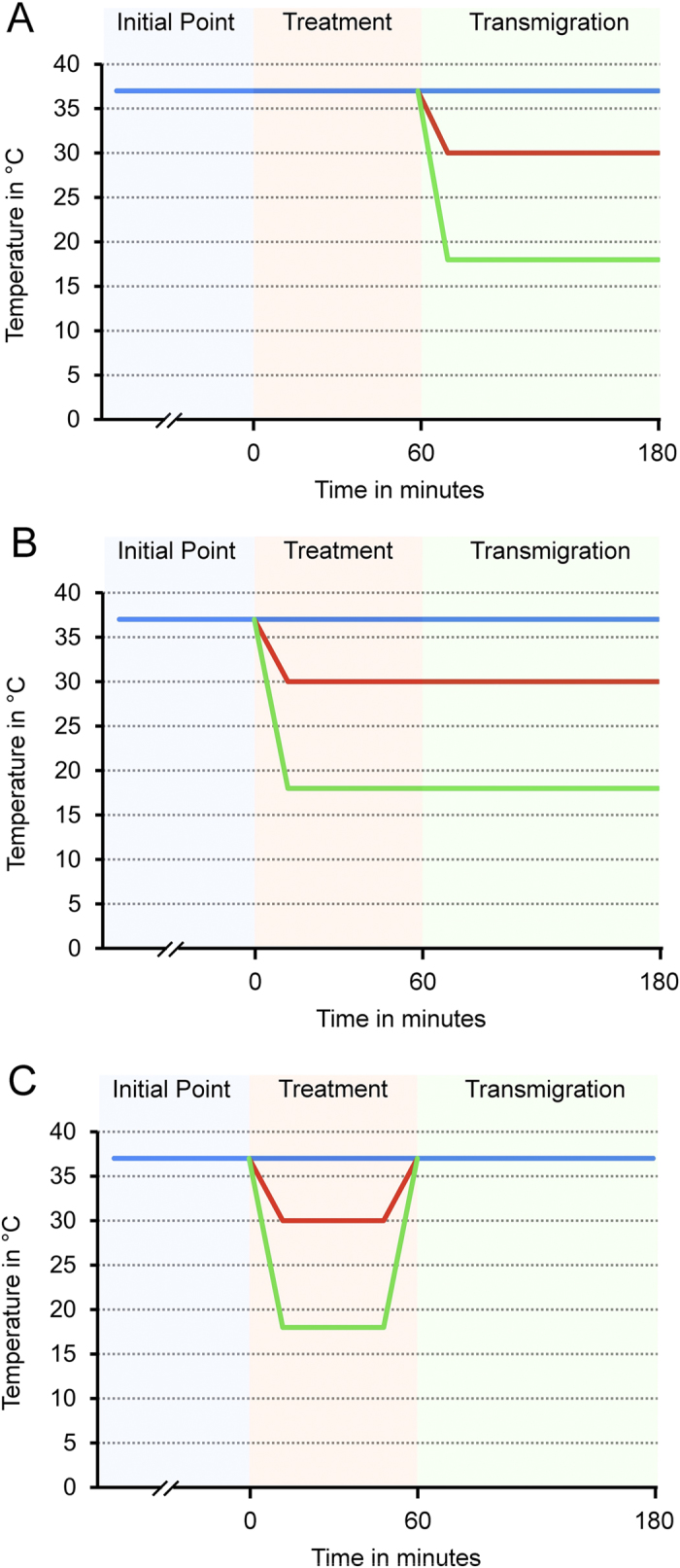
Scheme of experimental temperature modulation. Cells were kept at 37 °C initially. **(A)** HuMEC-1 and PBL were treated for 60 min at 37 °C. TEM was performed for 120 min at 37 °C, 30 °C and 18°. **(B)** HuMEC-1 and PBL were treated for 60 min at 37 °C, 30 °C and 18 °C. TEM was performed for 120 min under the same temperature conditions as HuMEC-1 and PBL were treated before. **(C)** HuMEC-1 and PBL were treated for 60 min at 37 °C, 30 °C or 18 °C with subsequent rewarming to 37 °C during TEM for 120 min.

**Figure 2:**
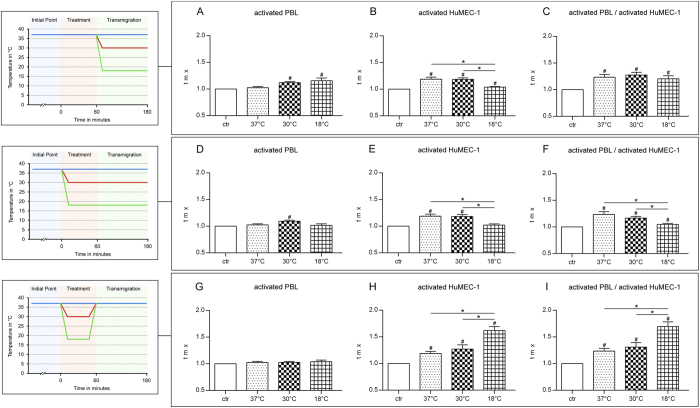
Influence of temperature modulation on transendothelial migration. (**A–C**) Influence on TEM when temperature modulation is only performed during transmigration in accordance to Fig. 1A. (**A**) Activated PBL show a significantly increased TEM with falling temperatures (37 °C n = 11, 30 °C n = 11, 18 °C n = 18). (**B**) TEM of non-activated PBL through activated HuMEC-1 is significantly reduced with deeper temperatures (37 °C n = 11, 30 °C n = 12, 18 °C n = 17), whereas (**C**) TEM of activated PBL through activated HuMEC-1 is not influenced with falling temperatures (37 °C n = 12, 30 °C n = 11, 18 °C n = 17). (**D–F**) Influence on TEM when temperature modulation is applied during treatment and transmigration in accordance to Fig. 1B. (**D**) TEM of activated PBL through non-activated HuMEC-1 is not significantly modulated during cooling (37 °C n = 11, 30 °C n = 11, 18 °C n = 12), whereas (**E**) TEM of non-activated PBL through activated HuMEC-1 is significantly reduced with falling temperatures (37 °C n = 11, 30 °C n = 12, 18 °C n = 12). (**F**) Cooling shows a significant protective function with reduced TEM of activated PBL through activated HuMEC-1 (37 °C n = 12, 30 °C n = 11, 18 °C n = 12). (**G–I**) Influence on TEM when temperature is modulated during treatment with following rewarming during transmigration in accordance to Fig. 1C. (**G**) Rewarming shows no influence on TEM of activated PBL through non-activated HuMEC-1 (37 °C n = 11, 30 °C n = 11, 18 °C n = 11). (**H**) TEM of non-activated PBL through activated HuMEC-1 is significantly enhanced with falling temperatures (37 °C n = 11, 30 °C n = 12, 18 °C n = 11). (**I**) Rewarming significantly enhances TEM of activated PBL through activated HuMEC-1 (37 °C n = 12, 30 °C n = 12, 18 °C n = 11). ^#^p < 0.05 vs. control, *p < 0.05.

In the first study-group, temperature modulation was performed as described in Fig. 1A. Results are summarized as transmigration-index (tmx) in Fig. 2A–C. Transmigration of activated PBL through non-activated HuMEC-1 under hypothermic conditions shows no significant changes with falling temperatures, whereas significant changes are noticeable compared to the control-group at 30 °C (p = <0.0001) and 18 °C (p = 0.0207). Hypothermic conditions during the transmigration of non-activated PBL through activated HuMEC-1 show significant changes in TEM with falling temperatures. Significant reductions of TEM are visible at 18 °C compared to 37 °C (p = <0.0001) and at 18 °C compared to 30 °C (p = <0.0001). Compared to the control-group, significant changes are observable at 37 °C (p = <0.0001), 30 °C (p = <0.0001) and 18 °C (p = 0.0049). The application of hypothermia during transmigration of activated PBL through activated HuMEC-1 causes no significant changes in TEM. Significant changes are noticeable compared to the control-group at 37 °C (p = 0.0002), 30 °C (p = <0.0001) and 18 °C (p = 0.0055).

#### Hypothermic treatment and migration reduces transendothelial migration significantly

In the second study-group, temperature modulation was performed as described in Fig. 1B. Results are summarized as transmigration-index (tmx) in Fig. 2D–F. Application of hypothermia during treatment and transmigration of activated PBL through non-activated HuMEC-1 causes no significant change in TEM within the temperature range, whereas significant changes are noticeable compared to the control-group at 30 °C (p = 0.0001). Temperature modulation during cell treatment and transmigration of non-activated PBL through activated HuMEC-1 leads to a significant reduction of TEM at 18 °C compared to 37 °C (p = 0.0003) and at 18 °C compared to 30 °C (p = 0.0003). Compared to the control-group, significant changes are visible at 37 °C (p = <0.0001) and 30 °C (p = <0.0001). Transmigration of activated PBL through activated HuMEC-1 is affected under mild and severe hypothermic conditions, with significant diminished TEM at 18 °C compared to 37 °C (p = 0.0003) and at 18 °C compared to 30 °C (p = 0.0472). Significant changes are noticeable compared to the control-group at 37 °C (p = 0.0002), 30 °C (p = <0.0001) and 18 °C (p = 0.0241).

#### Post-hypothermic rewarming strongly increases transendothelial migration

In the third study-group, temperature modulation was performed as described in Fig. 1C. Results are summarized as transmigration-index (tmx) in Fig. 2G–I. TEM of activated PBL through non-activated HuMEC-1 is not significantly affected after rewarming. Temperature modulation during treatment and following rewarming during transmigration of non-activated PBL through activated HuMEC-1 leads to a highly enhanced TEM with significant changes at 18 °C compared to 37 °C (p = 0.0003) and at 18 °C compared to 30 °C (p = 0.0035). Significant changes are noticeable compared to the control-group at 37 °C (p = <0.0001), 30 °C (p = 0.0018) and 18 °C (p = <0.0001). Simultaneous activation of PBL and HuMEC-1 leads to the same enhanced TEM in accordance to a growing temperature difference. Significant increases of TEM can be observed at 18 °C compared to 37 °C (p = <0.0001) and 18 °C compared to 30 °C (p = 0.0007). Compared to the control-group, significant changes are visible at 37 °C (p = 0.0002), 30 °C (p = 0.0015) and 18 °C (p = <0.0001).

### Surface-expression-assay

#### JAM-A-surface-expression is significantly modulated during post-hypothermic rewarming

**Figure 3:**
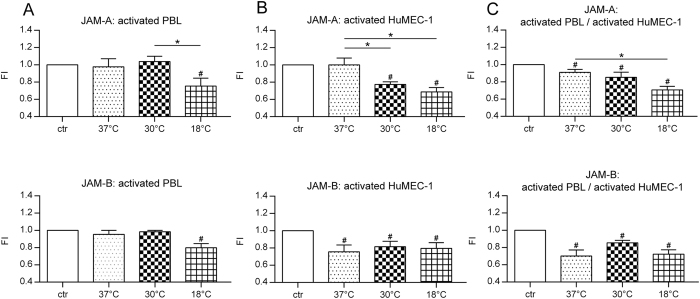
Modulation of JAM-A- and JAM-B-surface-expression during rewarming. **(A)** Co-culture of activated PBL and non-activated HuMEC-1 significantly decreases expression of JAM-A (37 °C n = 7, 30 °C n = 6, 18 °C n = 6) with falling temperatures. JAM-B (37 °C n = 8, 30 °C n = 6, 18 °C n = 5) shows no significant change during temperature modulation. **(B)** Rewarming in a co-culture of non-activated PBL and activated HuMEC-1 shows significantly decreased expression of JAM-A (37 °C n = 8, 30 °C n = 6, 18 °C n = 6) with growing temperature differences, whereas JAM-B is not affected (37 °C n = 8, 30 °C n = 6, 18 °C n = 6). **(C)** Co-culture with activated PBL and activated HuMEC-1 leads to a significant down-regulation of JAM-A expression (37 °C n = 8, 30 °C n = 6, 18 °C n = 6) with falling temperatures, whereas JAM-B expression is not affected (37 °C n = 8, 30 °C n = 6, 18 °C n = 6). ^#^p < 0.05 vs. control, *p < 0.05.

For surface-expression-assay, temperature modulation was performed as described in Fig. 1C and results of endothelial JAM-A-surface-expression are summarized as fluorescence-intensity (FI) in Fig. 3A–C. Post-hypothermic rewarming of activated PBL in a co-culture with non-activated HuMEC-1 leads to significant decreased JAM-A-surface-expression at 18 °C compared to 30 °C (p = 0.0474) and a significant change at 18 °C compared to the control-group (p = 0.0224). Activated HuMEC-1 in a co-culture with non-activated PBL show a significant decreased JAM-A-surface-expression at 30 °C compared to 37 °C (p = 0.0431) and at 18 °C compared to 37 °C (p = 0.0027). The control-group shows increased JAM-A-surface-expression compared to 30 °C (p = <0.0001) and to 18 °C (p = 0.0001). Co-culture of simultaneously activated HuMEC-1 and PBL leads to significant decreased JAM-A-surface-expression at 18 °C compared to 37 °C (p = 0.0039). Compared to the control-group, significant changes are visible at 37 °C (p = 0.0217), 30 °C (p = 0.0198) and 18 °C (p = 0.0001).

#### JAM-B-surface-expression is not significantly modulated during post-hypothermic rewarming

For surface-expression assay, temperature modulation was performed as described in Fig. 1C and results of endothelial JAM-B-surface-expression are summarized as fluorescence-intensity (FI) in Fig. 3. JAM-B-surface-expression is not significantly modulated within the applicated temperature-range and significant changes are only noticeable in comparison to the control-group. Activated PBL in a co-culture with non-activated HuMEC-1 leads to significant decreased JAM-B-surface-expression at 18 °C compared to the control-group (p = 0.0012). Activated HuMEC-1 in a co-culture with non-activated PBL show a significant decreased JAM-B-surface-expression compared to control at 37 °C (p = 0.0088), 30 °C (p = 0.0082) and 18 °C (p = 0.0108). Co-culture of simultaneously activated HuMEC-1 and PBL leads to significant decreased JAM-B-surface-expression compared to control at 37 °C (p = 0.0009), 30 °C (p = 0.0002) and 18 °C (p = 0.0003).

### Total-expression-assay

#### JAM-total-protein-expression is not significantly modulated during rewarming

**Figure 4:**
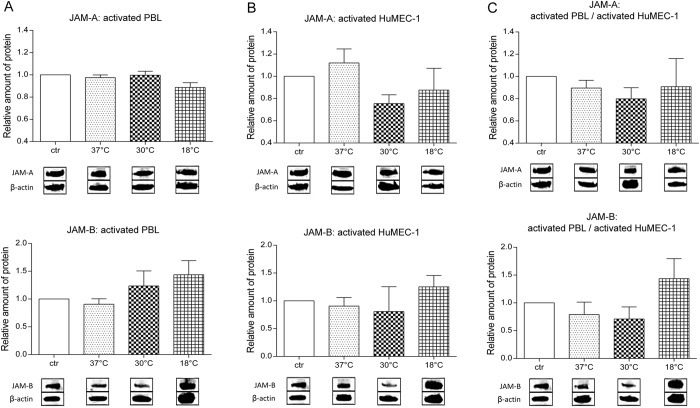
Modulation of JAM-A- and JAM-B-total-expression during rewarming. (**A)** Co-culture of activated PBL and non-activated HuMEC-1 shows no significant changes on expression of JAM-A (37 °C n = 2, 30 °C n = 2, 18 °C n = 2) and JAM-B (37 °C n = 2, 30 °C n = 2, 18 °C n = 2) with falling temperatures. **(B)** Rewarming in a co-culture of non-activated PBL and activated HuMEC-1 shows no significant influence on expression of JAM-A (37 °C n = 2, 30 °C n = 3, 18 °C n = 2) and JAM-B (37 °C n = 2, 30 °C n = 2, 18 °C n = 2). **(C)** Co-culture with activated PBL and activated HuMEC-1 leads to no significant regulation of JAM-A (37 °C n = 2, 30 °C n = 2, 18 °C n = 2) and JAM-B (37 °C n = 2, 30 °C n = 2, 18 °C n = 2) expression. ^#^p < 0.05 vs. control, *p < 0.05. Representative western-blots are presented for every experimental group and illustrate the JAM-A, JAM-B and β-actin-expression. Full-length blots are presented in Supplementary Figure 1.

For total-expression-assay, temperature modulation was performed as described in Fig. 1C and results of endothelial JAM-total-expression are summarized as relative amount of protein in Fig. 4. Neither JAM-A-total-expression nor JAM-B-total-expression is significantly modulated during post-hypothermic rewarming. As well no significant changes are noticeable compared to the control-group.

### Analysis of IL-6 and IL-10

#### Rewarming leads to significant modulation of IL-6 expression

**Figure 5:**
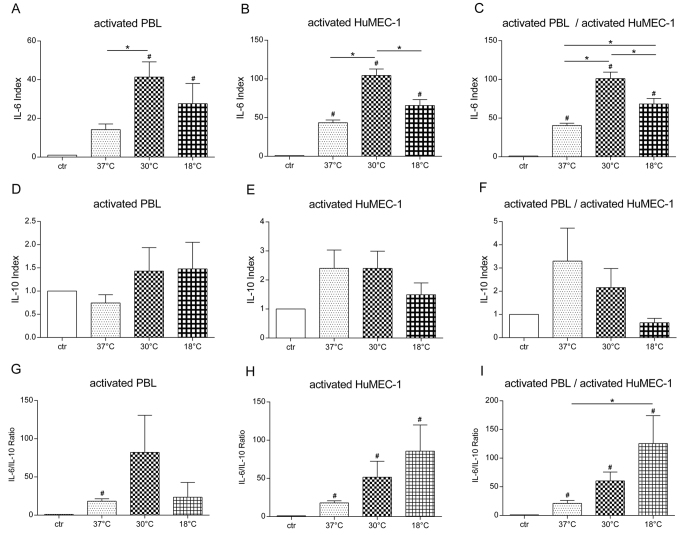
IL-6 and IL-10 during rewarming. (**A–C**) Influence of rewarming on IL-6. **(A)** Co-culture of activated PBL and non-activated HuMEC-1 shows significant influence on IL-6 (37 °C n = 5, 30 °C n = 5, 18 °C n = 3). **(B)** Rewarming in a co-culture of non-activated PBL and activated HuMEC-1 leads to a significant modulation of IL-6 with falling temperatures (37 °C n = 5, 30 °C n = 5, 18 °C n = 6). **(C)** This effect is also visible in a co-culture of activated PBL and activated HuMEC-1 (37 °C n = 6, 30 °C n = 5, 18 °C n = 5). **(D–F)** Influence of rewarming on IL-10. Temperature modulation shows no significant influence on IL-10 expression. **(D)** Co-culture of activated PBL and non-activated HuMEC-1 (37 °C n = 5, 30 °C n = 5, 18 °C n = 3). **(E)** Co-culture of non-activated PBL and activated HuMEC-1 (37 °C n = 5, 30 °C n = 5, 18 °C n = 6). **(F)** Co-culture of activated PBL and activated HuMEC-1 (37 °C n = 6, 30 °C n = 5, 18 °C n = 5). **(G–I)** Influence of rewarming on IL-6/IL-10 ratio. **(G)** Co-culture of activated PBL and non-activated HMEC-1 shows no significant changes in Il-6/IL-10 ratio (37 °C n = 5, 30 °C n = 5, 18 °C n = 3). (**H)** Co-culture of non-activated PBL and activated HuMEC-1 show no significant influence on IL-6/IL-10 ratio with falling temperatures (37 °C n = 5, 30 °C n = 5, 18 °C n = 6). **(I)** Co-culture with activated PBL and activated HuMEC-1 leads to significant up regulation of IL-6/IL-10 ratio (37 °C n = 6, 30 °C n = 5, 18 °C n = 5). ^#^p < 0.05 vs. control, *p < 0.05.

For analysis of IL-6 temperature modulation was performed as described in Fig. 1C and results are summarized in Fig. 5A–C. Activation of PBL in co-culture with non-activated HuMEC-1 leads to a significant up-regulation of IL-6 at 30 °C compared to 37 °C (p = 0.0431). Significant changes are visible at 30 °C (p = 0.002) and 18 °C (p = 0.0483) compared to control. Rewarming in a co-culture of non-activated PBL and activated HuMEC-1 causes a significant increase of IL-6 at 30 °C compared to 37 °C (p = <0.0001) and a significant decrease of IL-6 at 18 °C compared to 30 °C (p = 0.0008). Compared to the control-group significant changes are visible at 37 °C (p = 0.0003), 30 °C (p =<0.0001) and 18 °C (p = <0.0001). Co-culture of simultaneously activated HuMEC-1 and PBL leads to a significantly enhanced IL-6 expression at 30 °C (p = <0.0001) and at 18 °C (p = 0.0128) compared to 37 °C. Temperature modulation from 30 °C to 18 °C causes a significant decrease of IL-6 (p = 0.0031). Compared to the control-group, significant changes are visible at 37 °C (p = 0.0005), 30 °C (p = <0.0001) and 18 °C (p = <0.0001).

#### Post-hypothermic rewarming causes no significant changes of IL-10

For analysis of IL-10 temperature modulation was performed as described in Fig. 1C and results are summarized in Fig. 5D–F. Rewarming causes no significant changes of IL-10 with falling temperature conditions.

#### Post-hypothermic rewarming significantly raises the IL-6/IL-10 ratio

For analysis of IL-6/IL-10 ratio temperature modulation was performed as described in Fig. 1C and results are summarized in Fig. 5G–I. Activation of PBL in co-culture with non-activated HuMEC-1 leads to a significant change of IL-6/IL-10 ratio at 37 °C compared to control (p = 0.0002). Post-hypothermic rewarming in a co-culture of non-activated PBL and activated HuMEC-1 causes a significant increase of IL-6/IL-10 ratio compared to the control-group at 37 °C (p = <0.0001), 30 °C (p = 0.0249) and 18 °C (p = 0.0327). Co-culture of simultaneously activated HuMEC-1 and PBL leads to a significantly enhanced IL-6/IL-10 ratio at 18 °C compared to 37 °C (p = 0.0250). Compared to the control-group, significant changes are visible at 37 °C (p = 0.0028), 30 °C (p = 0.0023) and 18 °C (p = 0.0199).

## Discussion

Several studies have analyzed the single molecular effects of hypothermia or post-hypothermic rewarming during inflammatory processes, but the final common effects on leukocyte recruitment remain unclear. The current study investigates TEM of PBL through an endothelial monolayer in an *in-vitro* inflammatory model, using typical clinical temperature conditions. Additionally, we investigated the expression of JAM-A and JAM-B after post-hypothermic rewarming. Cooling of normothermic cells during cell activation and transmigration or the single application of hypothermia during transmigration reduces TEM and promotes an anti-inflammatory effect. Hypothermic cell activation and subsequent rewarming during transmigration leads to enhanced TEM of PBL through an endothelial monolayer and evokes a pro-inflammatory effect. Post-hypothermic rewarming modulates endothelial JAM-expression. Especially the down regulation of JAM-A expression could be associated with the enhanced TEM of PBL during the rewarming process.

It is well established that inflammatory processes induce several intracellular cascades and signaling pathways. One of the acting pathways during inflammation is the activation of NF-ĸB, which is associated with the expression of cytokines, chemokines or adhesion molecules^23^,^24^. Several studies have shown that NF-ĸB is modulated during hypothermic conditions^9^,^25^,^26^. Furthermore, some of them show that hypothermia leads to an activation of NF-ĸB^9^,^25^, whereas other studies show an inhibition of NF-ĸB^26^. The same variable effects of hypothermia on cellular modulation are controversial discussed in studies which analyzed cytokine expression^9^,^26^,^27^. Furthermore, the cells used in our study were stimulated concomitantly to hypothermia with LPS to mimic an inflammatory process, as would happen in a real clinical situation. Recently, Thiel *et al.*^28^ used a similar strategy to reproduce a pro-inflammatory status in a murine model of lupus nephritis. Hypothermia is able to modulate cytokine expression, which could lead to an anti-inflammatory^9^ or a pro-inflammatory effect^26^. Adhesion molecules play an essential role in initiating transendothelial migration. Typical adhesion molecules such as ICAM-1^14^, E-Selectin^15^, VCAM or ELAM^16^ show a decreased expression during hypothermic conditions. Down regulation of adhesion molecules could be associated with decreased TEM during hypothermic conditions. Seo *et al.*^27^ showed that long-term hypothermic conditions over 6 h are able to prevent microglial migration. Our study confirms this observation and illustrates the final common pathway of short-term hypothermia, which leads to decreased TEM of PBL through an endothelial monolayer. Short-term hypothermia applied during transmigration or during activation and transmigration may simultaneously lead to reduced leukocyte infiltration. This process depends on the depth of applied hypothermic conditions. Deeper temperature conditions lead to a higher inhibitory effect on TEM.

Clinical application of hypothermia requires as well rewarming processes to regain normothermic conditions. Several studies show that post-hypothermic rewarming modulates inflammatory responses. Not only hypothermic conditions are able to modulate NF-kB, but also post-hypothermic rewarming is able to modulate this essential inflammatory pathway^20^,^29^,^30^. Diestel *et al.*^29^ describe a diminished degradation of the inhibitor of regulation of NF-ĸB (IĸB-α) in the first 30 min during rewarming, followed of a delayed and higher degradation of IĸB-α after 50 min during the rewarming process compared to normothermic conditions. Other studies describe an augmentation of NF-ĸB during rewarming^20^ and show that NF-ĸB is up regulated on the first postoperative day after deep hypothermic conditions followed by fast rewarming compared to slow rewarming^30^. The effect of posthypothermic rewarming on cytokines is also controversially discussed, which could lead to a pro-inflammatory cytokine expression^19^,^31^ or to an anti-inflammatory cytokine expression^32^,^33^. Awad *et al.*^20^ show that posthypothermic rewarming causes an up regulation of typical adhesion molecules such as ICAM-1, E-Selectin and VCAM-1. Up regulation of adhesion molecules could be associated with higher transmigration potency during posthypothermic rewarming. Our study shows an increased TEM of PBL through an endothelial monolayer during post-hypothermic rewarming. This enhancement of TEM seems to be temperature dependent. As the temperature differences between initial-point, hypothermic conditions and rewarming grows, the leukocyte recruitment gets more essentially influenced.

We hypothesized that this increasing influence of rewarming is caused by regulation of JAMs. JAMs are as well modulated during inflammatory immune response and could be expressed through the activation of the typical inflammatory NF-ĸB pathway. Azari *et al.*^34^ and Ohkuni *et al.*^35^ show that inhibition of NF-ĸB resulted in a decreased JAM-A expression. In another study, JAM-A expression was decreased during induction of a pro-inflammatory response through an TLR3 ligand, whereas TNF-α and IL-8 expression increases. Both effects are mediated through NF-ĸB^35^. Comparing this report to the previous described changes, post-hypothermic rewarming could lead to a pro-inflammatory response with enhanced cytokine production and reduced JAM-A expression. Laukoetter *et al.*^36^ observed that JAM-A deficient animals suffer from a breakdown of intestinal barrier function, associated with higher leukocyte recruitment and increased permeability. Our data suggest that decreased JAM-A expression leads to higher leukocyte recruitment and show that posthypothermic rewarming leads to a decreased JAM-A expression in a temperature dependent manner. JAM-B expression is less modulated during temperature changes and shows no correlation with TEM. Temperature shift during rewarming after short-term hypothermia causes a breakdown of endothelial barrier function and leads to higher TEM of PBL.

The *in vitro* re-enactment of clinical practice has shown a significant influence of temperature modulation on TEM. Our data show that TEM of PBL through endothelial cells is not only modulated by cell-activation itself - the activation temperature and the rewarming processes are essential. Prolonged hypothermia without rewarming is able to inhibit TEM, whereas hypothermia with post-hypothermic rewarming enhances TEM strongly, mainly depending on the difference between hypothermic temperature and rewarming temperature, with increased inflammatory activation in higher temperature differences. The IL-6/IL-10 ratio clearly shows an increased pro-inflammatory status depending on the delta between the target hypothermia temperature and 37 °C. The modifications in JAMs, especially JAM-A, provide a potential mechanistic explanation for the observed changes in TEM during the rewarming process. Endothelial protection prior to warm reperfusion and mild hypothermic conditions should be considered in clinical practice.

## Methods

### Ethics Statement

This study was approved by the ethics committee of the University of Frankfurt, Germany (GN.:227/13). All blood samples were obtained under informed consent and according to the declaration of Helsinki. All participants provided their written informed consent to participate in this study.

### Cell culture

Human microvascular endothelial cells-1 (HuMEC-1) were kindly provided by Dr. V. Mirakaj (University Tübingen, Department of Anesthesiology and Intensive Care Medicine) and cultured in MCDB-131 (Life Technologies, Darmstadt, Germany) supplemented with 10% fetal calf serum (Gibco, Karlsruhe, Germany), 1% glutamine (Gibco, Karlsruhe, Germany), 1% pen/strep solution (Sigma Chemical Co. St. Louis, USA), 10 ng/ml Epidermal Growth Factor (Sigma Chemical Co. St. Louis, USA) and 1 μg/ml hydrocortisone (Sigma Chemical Co. St. Louis, USA) at 37 °C and 5% CO_2_ atmosphere.

### Isolation of peripheral blood leukocytes (PBL)

Blood was drawn into EDTA tubes from healthy male and female volunteers (n = 5). For density gradient centrifugation, the fresh blood samples were underlaid with 5 ml Polymorphprep® (Axis-Shield, Oslo, Norway) and centrifuged at 450 g for 35 min. The layers of peripheral blood leukocytes (PBL), containing peripheral blood mononuclear cells (PBMC) and polymorph nuclear cells (PMNC)^37^, were aspirated and washed with PBS (Gibco, Karlsruhe, Germany). PBL were suspended in 37 °C pre-warmed RPMI 1640 (Life Technologies, Darmstadt, Germany).

### Influence of temperature modulation on transendothelial migration

#### Cultivation of an endothelial monolayer on cell culture inserts

Transmigration assays were performed with the use of 3-μm pore size FluoroBlok inserts (BD, Heidelberg, Germany) in triplicate. BrdU-labeled non-proliferating HuMEC-1 were placed on fibronectin-coated inserts and were allowed to form an endothelial monolayer for approximately 24 h at 37 °C, 5% CO.

#### Treatment of cultivated HuMEC-1

As previously described^37^, HuMEC-1 were partially activated with 100 μg/ml LPS (Sigma Chemical Co. St. Louis, USA) in MCDB-131 (Life Technologies, Darmstadt, Germany), whereas the other fraction of HuMEC-1 remained non-activated. The treatment of every study group was performed for 60 min under different temperature conditions (Fig. 1A–C). After treatment, medium was removed and transmigration-assay was started.

#### Treatment of PBL

PBL were activated with 10 μg/ml LPS in RPMI 1640 with 10% fetal bovine serum (Gibco, Karlsruhe, Germany), whereas the other fraction of PBL remained non-activated. Treatment of every study group was performed for 60 min under different temperature conditions (Fig. 1A–C). Cells were washed after incubation with PBS.

#### Study groups

Following subassembly-groups were used within the main-groups: A) non-activated PBL and non-activated HuMEC-1 (control); B) activated PBL and non-activated HuMEC-1; C) non-activated PBL and activated HuMEC-1; D) activated PBL and activated HuMEC-1. Temperature modulation (Fig. 1) was performed to every subassembly group in the same manner, whether if they were activated or remained non-activated. In the first main-group (Fig. 1A), temperature modulation (37 °C, 30 °C and 18 °C) was only performed during transmigration. In the second main-group (Fig. 1B), temperature modulation was performed during treatment and during transmigration. In the third main-group (Fig. 1C), temperature modulation was only performed during treatment following normothermic transmigration, which mimics the posthypothermic rewarming process. The control group consisted in all cases of non-activated PBL and non-activated HuMEC-1 undergoing the same temperature modulation strategies as the study groups.

#### Transmigration-assay

PBL stained with Calcein-AM (ABD-Bioquest, Biomol, Hamburg, Germany) were suspended in assay-medium (RPMI-1640 + 0.5% BSA) and applied on top of the endothelial monolayer. The transmigration assay was performed for 120 min under normothermic and hypothermic conditions (37 °C, 30 °C, 18°). Transmigration conditions of all three main-groups are shown in Fig. 1A–C. The fluorescence signal of transmigrated PBL to the bottom surface of the membrane insert was measured with a microplate reader (Berthold Technologies, Bad Wildbad, Germany) at 485/535 nm. Transmigrated cells are expressed as percent of control (transmigration index, tmx).

### JAM-A- and JAM-B-surface-expression during rewarming

#### Cultivation of HuMEC-1

Surface-expression-assay was performed with the use of black 96-Well-Plates with clear-bottom (BD, Heidelberg, Germany). BrdU-labeled non-proliferating HuMEC-1 were placed on fibronectin-coated wells and were allowed to form an endothelial monolayer for approximately 24 h at 37 °C, 5% CO.

#### Treatment of cells and Co-culture conditions

HuMEC-1 and PBL were treated as previously described. Normothermic cells were treated at different temperature conditions (37 °C, 30 °C, 18 °C) for 60 min (Fig. 1C). Activated or non-activated HuMEC-1 and activated or non-activated PBL were incubated together in a co-culture with transmigration-medium (RPMI-1640 + 0.5% BSA). Co-culture was performed at 37 °C for 120 min to simulate the rewarming process (Fig. 1C). After incubation, PBL were removed and HuMEC-1 were washed with PBS.

#### Immunofluorescence analysis

HuMEC-1 were fixed with 4% PFA (AppliChem, Darmstadt, Germany) at RT and were blocked with PBS supplemented with 10% donkey serum (Jackson ImmunoResearch Europe Ltd., Suffolk, UK). HuMEC-1 were primarily stained with rabbit anti-JAM-A-IgG [1:200] (Santa Cruz Biotechnology, Heidelberg, Germany) and goat anti-JAM-B-IgG [1:100] (Santa Cruz Biotechnology, Heidelberg, Germany). Secondary antibody incubation was performed with donkey anti-rabbit-IgG-PE [1:100] (Santa Cruz Biotechnology, Heidelberg, Germany) or donkey anti-goat-IgG-FITC [1:100] (Santa Cruz Biotechnology, Heidelberg, Germany) in PBS containing 1% donkey serum. JAM-surface-protein expression was measured with a microplate reader (Berthold Technologies, Bad Wildbad, Germany) at 485/535 nm and 530/590 nm. Fluorescence intensity (FI) was calculated in relation to control.

### JAM-A- and JAM-B-total-expression during rewarming

#### Cultivation of HuMEC-1

Total-expression-assay was performed with the use of T25 flasks (Sarstedt, Nürmbrecht, Germany) and HuMEC-1 were allowed to form an endothelial monolayer for approximately 24 h at 37 °C, 5% CO.

#### Treatment of cells and Co-culture conditions

HuMEC-1 and PBL were treated in the same manner than previously described in immunofluorescence-analysis of surface-expression.

#### Protein isolation and Western Blot Analysis

Cells were harvested with cell scrapers and proteins were isolated from cell pellet under constant agitation with cell lysis buffer for 10 min at 4 °C. Cell lysates were applied to a 4–20% Mini-Protean TGX gel (BioRad, München. Germany) and electrophoresis was applied at 200 V for 20 min. Proteins were transferred to PVDF-membranes (BioRad, München. Germany) at 2,5 mA for 5 min. Membranes were blocked for 60 min with blocking-buffer containing 5% serum (Jackson ImmunoResearch Europe Ltd., Suffolk, UK) of the species where the second antibody was raised in. Primary antibody incubation of rabbit anti-JAM-A-IgG [1:200] (Santa Cruz Biotechnology, Heidelberg, Germany) and goat anti-JAM-B-IgG [1:200] (Santa Cruz Biotechnology, Heidelberg, Germany) was performed over night at 4 °C. Secondary antibody incubation of donkey anti-goat-IgG-HRP [1:5000] (Santa Cruz Biotechnology, Heidelberg, Germany) and goat anti-rabbit-IgG-HRP [1:5000] (Merck, Darmstadt, Germany) was performed for 60 min at RT. Membranes were incubated with HRP substrate solution (Merck, Darmstadt, Germany) and proteins were visualized with Fusion FX7 system (Peqlab, Erlangen, Germany). β-Actin was used as an internal control for protein loading. The visualized protein bands were analyzed using ImageJ. The relative amount of protein was calculated in relation to β-actin expression and to control-group. All gels were ran under the same experimental conditions. Cropped western-blots are presented in Fig. 4 and full-length blots are presented in supplementary Figure 1.

### Analysis of IL-6 and IL-10 during rewarming

#### Collection of Co-culture supernatants and ELISA procedure

Co-culture supernatants from western blot analysis were collected before cells were harvested. Supernatants were centrifuged to remove the PBL cell pellet. IL-6 (R&D, Abingdon, Great Britain) and IL-10 (R&D, Abingdon, Great Britain) ELISA were performed in accordance to the manufacturers guide. IL-6 and IL-10 are expressed as percent of control (IL-6 Index, IL-10 Index) or were evaluated in relation to each other to calculate an IL-6/IL-10-Ratio.

### Statistical analysis

All data represent mean ± standard error of the mean (SEM). Statistical analysis was performed with Prism 6 software (Graph Pad, La Jolla, USA) using Analysis of Variance (ANOVA) and *post-hoc* Tukey’s-multiple-comparisons-test. Differences with p < 0.05 were considered statistically significant.

## Additional Information

**How to cite this article**: Bogert, N. V. *et al.* Influence of hypothermia and subsequent rewarming upon leukocyte-endothelial interactions and expression of Junctional-Adhesion-Molecules A and B. *Sci. Rep.* **6**, 21996; doi: 10.1038/srep21996 (2016).

## Supplementary Material

Supplementary Information
