# Supplementary material for: Influence of hypothermia and subsequent rewarming upon leukocyte-endothelial interactions and expression of Junctional-Adhesion-Molecules A and B
Source: Sci Rep. 2016 Feb 25;6:21996. doi: 10.1038/srep21996 (PMC4766492; doi:10.1038/srep21996)
Supplement: Supplementary Information [file srep21996-s1.doc]

**Influence of hypothermia and subsequent rewarming upon leukocyte-endothelial interactions and expression of Junctional-Adhesion-Molecules A and B**

Nicolai V. Bogert^1^, Isabella Werner^1^ PhD, Angela Kornberger^1^ MD, Patrick Meybohm^2^MD, Anton Moritz^1^ MD, Till Keller^3^ MD, Ulrich A. Stock^1^ MD, Andres Beiras-Fernandez^1,*^ MD, PhD

^1^ Department of Thoracic and Cardiovascular Surgery, University Hospital Frankfurt, Goethe University, Frankfurt/Main, Germany.^2^ Department of Anaesthesiology, University Hospital Frankfurt, Goethe University, Frankfurt/Main, Germany.^3^ Department of Cardiology, University Hospital Frankfurt, Goethe University, Frankfurt/Main, Germany.

**Authors Contribution Statement**

**Manuscript**

Influence of hypothermia and subsequent rewarming upon leukocyte-endothelial interactions and expression of Junctional-Adhesion-Molecules A and B

**Authors**

Nicolai V. Bogert & Isabella Werner PhD: Conducted experiments; Analyzed Data, Prepared Draft

Angela Kornberger MD: Analyzed Data; Reviewed Manuscript

Patrick Meybohm MD: Analyzed Data; Reviewed Manuscript

Anton Moritz MD: Analyzed Data; Reviewed Manuscript

Till Keller MD: Analyzed Data; Reviewed Manuscript

Ulrich A. Stock MD: Analyzed Data; Reviewed Manuscript

Andres Beiras-Fernandez MD, PhD: Designed Experiments; Analyzed Data; Prepared Draft, Reviewed Manuscript

**AB-F and IW contributed equally to this Manuscript**

**

**
